# The Relationship Between Pre-Loss Grief, Preparedness and Psychological Health Outcomes in Relatives of People With Cancer

**DOI:** 10.1177/00302228221142675

**Published:** 2022-11-24

**Authors:** Viktoria Schmidt, Julia Kaiser, Julia Treml, Anette Kersting

**Affiliations:** 1 Department of Psychosomatic Medicine and Psychotherapy, 70622 University of Leipzig, Leipzig, Germany

**Keywords:** anticipatory grief/mourning, grief, bereavement, death, dying

## Abstract

The aim of this study was to examine the simultaneous effects of pre-loss grief, preparedness for death and preparedness for caregiving on different psychological health outcomes in relatives of people with cancer. Two hundred ninety-nine relatives of people with cancer participated in a cross-sectional online survey. Participants were included if they spoke German and were 18 years or older. Multivariate regression analysis was conducted. Pre-loss grief was significantly associated with depression (β = .388, *p* < .001), anxiety (β = .429, *p* < .001), somatization (β = .221, *p* < .001) and satisfaction with life (β = −.205, *p* < .001). Preparedness for death was significantly associated with somatization (β = −.247, *p* < .001). Results suggest that people with high scores in pre-loss grief and low scores in preparedness for death are in need of early support. Interventions should address pre-loss grief and the various aspects of preparedness for death and take into account the psychological health in relatives of people with cancer. Future studies should investigate underlying mechanisms.

Cancer is a common cause of death for people worldwide, resulting in almost 10 million deaths in the year 2020 (Sung et al., 2021). Cancer frequently has a progressive course and in contrast to other causes of death, most relatives state that they had expected the death of the person with cancer (Caserta et al., 2019). This period of awaiting can give relatives time to adjust to the approaching loss and is described as pre-loss grief or anticipatory grief and preparedness in research (Nielsen et al., 2016; Treml et al., 2021). Preparedness can be further divided into preparedness for caregiving and preparedness for death, with the former having a validated scale that has been widely used in studies (Pucciarelli et al., 2014).

All three constructs seem to have an influence on pre-loss psychological health and the adjustment period after a loss in relatives of people with cancer (Treml et al., 2021). Literature on the effects of pre-loss grief or preparedness on relatives’ health found that higher levels of pre-loss grief were associated with a greater likelihood of current or past depressive or anxiety symptoms in relatives of people with cancer (Treml et al., 2021). On the other hand, high levels of preparedness for death and preparedness for caregiving seem to be related to lower depressive and anxiety symptoms (Caserta et al., 2019; Henriksson & Arestedt, 2013; Winterling et al., 2021).

Moreover, satisfaction with life was found to be negatively related to pre-loss grief and positively related to preparedness for caregiving (Holley, 2009; Ostwald et al., 2009; Walker & Pomeroy, 1997). The relationship between preparedness for death and satisfaction with life was only studied in one study, which found no significant association between these two constructs (Kim et al., 2015). However, preparedness for death was examined retrospectively, therefore possibly biasing the results.

Furthermore, Lindemann (1944) suggested, that anticipatory grief is characterized by somatic distress. While previous studies found a negative relationship between pre-loss grief and physical health (Garand et al., 2012; Walker & Pomeroy, 1997), the specific relationship between preparedness for death or preparedness for caregiving and somatization has not been examined yet. However, low levels of preparedness for death have been found to be associated with an increased risk for chronic pain after the death of a loved one for young widowers (Ásgeirsdóttir et al., 2013). Preparedness for caregiving has shown a positive association with physical health quality of life (Zale et al., 2018).

While previous studies suggest that pre-loss grief and preparedness have an effect on relatives’ health outcomes, findings have to be seen in light of some limitations. Preparedness for death has often been examined with a single question in previous studies, although a multidimensional concept with cognitive, behavioral, and affective facets was already proposed in 2009 by Hebert et al. (2009). First attempts of a multidimensional operationalization can be found by Caserta et al. (2019) who, however, only considered two of the aforementioned three facets. Further, the relationship to pre-loss health outcomes has been studied only to a limited extent. Previous studies have mostly examined either pre-loss grief or preparedness and have not distinguished between the two different forms of preparedness. However, to better understand the impact of the forewarning period on relatives’ health, it is necessary to distinguish between the different constructs and examine them simultaneously.

Therefore, this study set out to investigate the simultaneous effects of pre-loss grief and preparedness on different health outcomes of relatives of people with cancer. Specifically, the aims were to examine the relationship between depressive symptoms, anxiety symptoms, somatization and satisfaction with life with (1) pre-loss grief, (2) preparedness for death and (3) preparedness for caregiving.

To our knowledge, this is the first study to examine the effects of pre-loss grief, preparedness for death and preparedness for caregiving simultaneously on different health outcomes in relatives of people with cancer. Identifying the differential effects of these constructs on various health outcomes may provide insights into the time before the loss of a loved one and thus contribute knowledge for future intervention opportunities for relatives.

## Method

### Procedure

The study was approved by the Ethics Committee and performed in accordance with the Declaration of Helsinki. Recruitment of participants took place from August 2020 to September 2021 through social media and healthcare professionals (e.g., through hospices, facebook help groups and counseling centers for cancer). Recruited were all people who had a relative with cancer. Relatives of people with cancer interested in the study had access to the study information by downloading or reading through the information. They were further provided a written consent form on the first page of the survey, followed by several self-report forms.

### Participants

A total of 646 participants showed interest in the study and provided written informed consent. Of these, 273 dropped out during the beginning of the online survey. Another 74 participants dropped out during the course of the study, yielding a total sample of *n* = 299 participants. Only subjects who had completed all questionnaires described below were included. To motivate participants, they had the chance to win one of 10 book vouchers worth 10 euros. To be eligible for this cross-sectional study, participants had to be at least 18 years old, speak German, be a relative of a person with cancer and provide written informed consent.

### Measures

#### Predictor variables

To address the limitations of previous studies, this study used the validated Caregiver Grief Scale (Meichsner et al., 2016) to measure pre-loss grief. Eleven items can be rated on a 5-point Likert-scale (1 = strongly disagree; 5 = strongly agree). A higher total score represents higher pre-loss grief. In the present study, Cronbach’s Alpha for pre-loss grief was good (α = .82).

For preparedness for caregiving, the Preparedness for Caregiving scale was used (Pucciarelli et al., 2014). Eight items can be rated on a 5-point Likert scale (0 = not at all prepared; 4 = very well prepared). A higher total score indicates a higher preparedness for caregiving. Cronbach’s Alpha in the present study was excellent for this scale (α = .91).

Instead of using a single item, preparedness for death was assessed with a multidimensional concept. Preparedness for death was assessed with a self-generated scale with three items based on Schulz et al. (2015) and Hebert’s et al. suggested multidimensional conceptualization (2009). Participants are asked to rate how prepared they feel on a 4-point Likert scale (1 = not at all; 4 = very, see Supplementary Material S1). A higher total score indicates a higher preparedness for death. Internal consistency for this scale was questionable in this study (α = .69). However, because the classification of internal consistency varies widely among studies (see Taber, 2018) and internal consistency higher than .6 or .7 has been characterized as “acceptable” (e.g., van Griethuijsen et al., 2015), the total scale was included in the analysis.

#### Outcome variables for psychological health outcomes

Depression was assessed using the depression module of the Patient Health Questionnaire (PHQ-9, Kroenke et al., 2001), consisting of nine items with response options between 0 (=not at all) and 3 (=nearly every day). Items can be added to obtain a total severity score for depression, which can range from 0 to 27 and can be interpreted in terms of depression severity from minimal to severe depression (0–4 = minimal depression, 5–9 = mild depression, 10–14 = moderate depression, 15–19 = moderately severe depression, 20–27 = severe depression). A higher score indicates a higher depression severity. Internal consistency for the total scale was good (α = .87) in this study.

Somatization was measured with the somatic symptom module of the Patient Health Questionnaire (PHQ-15, Kroenke et al., 2002) consisting of 15 items. For this study, participants were asked to answer a 4-point Likert scale (0 = not at all; 3 = nearly every day). Items can be added to a total somatic symptom scale, with higher values indicating higher somatic symptom severity. Cronbach’s Alpha for the somatic symptom scale was acceptable (α = .78) in this study.

Anxiety was measured with the Generalized Anxiety Disorder Scale (GAD-7, Spitzer et al., 2006). Seven items can be answered on a 4-point Likert-scale (0 = not at all; 3 = nearly every day). A higher overall score implies a higher anxiety level and can be interpreted in terms of severity of anxiety, which can range from minimal to severe anxiety (0–4 = minimal anxiety, 5–9 = mild anxiety, 10–14 = moderate anxiety, 15–21 = severe anxiety). The internal consistency of the GAD-7 scale was excellent (α = .90) in this study.

Satisfaction with Life was assessed using the Satisfaction with Life Scale (Diener et al., 1985) containing five items. These can be rated on a 7-point Likert scale (1 = strongly disagree; 7 = strongly agree). A higher total sum score indicates higher satisfaction with life and can be interpreted from extremely dissatisfied to extremely satisfied (5–9 = extremely dissatisfied, 10–14 = dissatisfied, 15–19 = slightly dissatisfied, 20 = neutral, 21–25 = slightly satisfied, 26–30 = satisfied, 31–35 = extremely satisfied). Cronbach’s Alpha for the total scale was excellent (α = .90) in this study.

### Statistical Methods

Analyses were conducted with the Statistical Package for Social Sciences, version 27 (IBM® SPSS®). To examine the effect of pre-loss grief and preparedness on psychological health, multivariate linear regression analyses with depression, anxiety, somatization and satisfaction with life as dependent variables were conducted. Age and gender of the relatives of patients with cancer were included as control variables. Because there were only two gender diverse participants, female and gender diverse participants were grouped together. Coefficients are reported with a 95% confidence interval. Because four different models were tested, we applied Bonferroni correction to account for multiple comparisons and statistical significance was therefore set at *p* < .0125.

## Results

### Sample Description

Most participants were women (90.3%), married (55.9%), had a high education (at least 12 years, 62.2%), a German citizenship (96.3%) and were religious (57.9%). On average, they were 41 years old (*M* = 41.35, *SD* = 12.21, Range: 18–78). In terms of relationship, most relatives were children (42.1%), partners (26.8%) or parents (11.0%) of a person with cancer. Regarding the cancer patients, 53.5% were women and the average age was 54 years (*M* = 54.25, *SD* = 19.97, Range: 0–96).

Completers and dropouts did not differ in age, school education and marital status. However, they significantly differed in religion, gender and degree of kinship to the diseased. A detailed description can be found in Table S2 in the Supplementary Material. Tables S3 and S4 of the Supplementary Material presents information on the type of cancer and subjective prognosis of participants’ loved ones.

Regarding the clinical characteristics of the sample, 51.1% of the sample showed minimal to mild depressive symptoms, while 24.1% reported to have moderate, 16.4% moderate severe and 8.4% severe depressive symptoms. Similarly, 46.2% percent showed minimal to mild anxiety, while 25.4% reported moderate and 28.4% severe symptoms. Regarding satisfaction with life, the majority reported to be slightly to extremely satisfied with their life (59.3%). Nevertheless, 9.4% reported to be extremely dissatisfied with their life. Moreover, a detailed description of the sample can be found in Table 1.

**Table 1.** Demographic and Clinical Characteristics of the Total Sample (*n* = 299).

Variable	*M* (*SD*)/*n* (%)	Scale range
Age	41.35 (12.21)	
Gender		
Female	270 (90.3)	
Male	27 (9.0)	
Diverse	2 (0.7)	
School education		
Low	14 (4.7)	
Medium	93 (31.1)	
High	186 (62.2)	
Missings	6 (2.0)	
Degree of kinship to person with cancer		
Relatives are		
Children	126 (42.1)	
Partners	80 (26.8)	
Parents	33 (11.0)	
Siblings	21 (7.0)	
Friends	11 (3.7)	
Other	28 (9.4)	
Age cancer patient	54.25 (19.97)	
Gender cancer patient		
Female	160 (53.5)	
Male	138 (46.2)	
Diverse	1 (0.3)	
Pre-loss grief	3.49 (0.77)	1–5
Preparedness for death	5.96 (2.19)	3–12
Preparedness for caregiving	15.10 (7.23)	0–32
Depression	9.98 (5.98)	0–27
Minimal	59 (19.7)	
Mild	94 (31.4)	
Moderate	72 (24.1)	
Moderate severe	49 (16.4)	
Severe	25 (8.4)	
Anxiety	10.79 (5.50)	0–21
Minimal	37 (12.4)	
Mild	101 (33.8)	
Moderate	76 (25.4)	
Severe	85 (28.4)	
Somatization, *M (SD)*	9.40 (5.41)	0–39
Satisfaction with life, *M (SD)*	21.45 (7.60)	5–35
Extremely dissatisfied, *n* (%)	28 (9.4)	
Dissatisfied, *n* (%)	26 (8.7)	
Slightly dissatisfied, *n* (%)	55 (18.4)	
Neutral, *n* (%)	13 (4.3)	
Slightly satisfied, *n* (%)	72 (24.1)	
Satisfied, *n* (%)	77 (25.8)	
Extremely satisfied, *n* (%)	28 (9.4)	

### Pre-Loss Grief, Preparedness and Psychological Health Outcomes

The analysis yielded four significant prediction models. Results show that depression (first model) was significantly associated with pre-loss grief (β = .388, *p* < .001). Moreover, anxiety (second model) was significantly associated with pre-loss grief (β = .429, *p* < .001). Somatization (third model) was significantly associated with pre-loss grief (β = .221, *p* < .001) and preparedness for death (β = −.247, *p* < .001). Lastly, satisfaction with life (fourth model) was significantly associated with pre-loss grief (β = −.207, *p* < .001).

Preparedness for death was not significantly associated with depression, anxiety and satisfaction with life. Moreover, preparedness for caregiving was not a significant correlate for any of the outcomes (see Table 2).

**Table 2.** Multivariate Regression Analysis.

	Depression		Anxiety		Somatization		Satisfaction with Life	
Model term	β (95% CI)	*p*-value	β (95% CI)	*p*-value	β (95% CI)	*p*-value	β (95% CI)	*p*-value
Intercept	(−5.175 to 4.219)	.841	(−3.330 to 5.040)	.688	(−.192 to 10.024)	.059	(17.844 to 30.411)	<.001
Age	.074 (−.018 to .091)	.188	−.015 (−.055 to .042)	.788	.125 (.007 to .125)	.028	−.035 (−.094 to .051)	.558
Female or diverse gender	−.015 (−2.528 to 1.900)	.780	.042 (−1.175 to 2.770)	.427	.135 (.632 to 5.447)	.014	.102 (−.249 to 5.673)	.072
Pre-loss grief	.388 (2.151 to 3.901)	<.001	.429 (2.291 to 3.851)	<.001	.221 (.902 to 2.805)	<.001	−.207 (−3.224 to −.882)	<.001
Preparedness for death	−.078 (−.558 to .131)	.224	−.044 (−.417 to .197)	.480	−.247 (−1.100 to −.351)	<.001	.078 (−.190 to .732)	.249
Preparedness for caregiving	−.005 (−.100 to .092)	.936	−.051 (−.124 to .047)	.378	.022 (−.085 to .124)	.712	.082 (−.043 to .215)	.190

*Note. N* = 299; Model 1-Depression: *R*^2^ = .17, Adjusted *R*^2^ = .16, *F*(5,293) = 11.970 (*p* < .001); Model 2-Anxiety: *R*^2^ = .22, Adjusted *R*^2^ = .21, *F*(5,293) = 16.528 (*p* < .001); Model 3-Somatization: *R*^2^ = .16, Adjusted *R*^2^ = .14, *F*(5,293) = 10.374 (*p* < .001); Model 4-Satisfaction with Life: *R*^2^ = .08, Adjusted *R*^2^ = .06, *F*(5,293) = 5.038 (*p* < .001).

## Discussion

This is, to our knowledge the first study to simultaneously investigate the association between pre-loss grief, preparedness for death and preparedness for caregiving on different psychological health outcomes in relatives of people with cancer. Results revealed that higher preparedness for death was associated with lower levels of somatization. Previous studies did not examine this specific relationship, but Ásgeirsdóttir et al. (2013) found that young widowers with low levels of preparedness for death were at an increased risk for chronic pain. Our findings suggest that the association between preparedness for death and somatization may occur regardless of age and thus the individual facets of preparedness for death should be considered prior to the loss of a loved one. Also, results indicate that gender may play a role in somatization, however, studies with a more diverse distribution of gender are necessary to explore the relationship between gender and somatization.

Regarding the relationship between preparedness for death and satisfaction with life, our results are in line with Kim et al. (2015), who also did not find a significant relationship between these constructs. A new finding of this study is that preparedness for death did not emerge as a significant correlate for depression and anxiety. Our results are contrary to a previous study that found a significant relationship between preparedness for death and depression or anxiety (Caserta et al., 2019). However, Caserta et al. (2019) assessed preparedness for death retrospectively, which could have led to recall bias and may account for the described differences. While preparedness for death may not be immediately related to relatives’ depression and anxiety before the loss, relatives may experience more depression and anxiety after the loss of a loved one, when they did not feel prepared for the death. Further research is necessary to investigate these effects in more detail.

Moreover, results showed that higher levels in pre-loss grief were significantly associated with more depressive, anxiety and somatic symptoms and a lower level of satisfaction with life. This is in accordance with previous findings on the negative relationship between pre-loss grief and relatives’ health (Garand et al., 2012; Holley, 2009; Treml et al., 2021; Walker & Pomeroy, 1997) and underlines the importance of support for relatives who are confronted with an impending loss of a loved one.

Our results further reveal that preparedness for caregiving did not emerge as a significant correlate among all outcomes. This could be due to the fact that 37% of the relatives reported that their loved one did not need any support or care from them. In contrast, the previously mentioned studies on preparedness for caregiving referred to samples in which relatives were taking care of their loved one, for example, as palliative caregivers, or when outpatient care was necessary for several months (Henriksson & Arestedt, 2013; Winterling et al., 2021). Therefore, it would be interesting to investigate whether the same results are obtained in caregiving relatives.

Results suggest that pre-loss grief emerges as a constant correlate for psychological health outcomes. Therefore, early support addressing not only pre-loss grief, but also symptoms of depression, anxiety and somatization as well as satisfaction with life for people in need is necessary. Furthermore, since preparedness for death and somatization seem to be associated, relatives should receive additional help to prepare for the impending death. To provide tailored interventions, further research on underlying mechanisms is necessary.

### Limitations

The findings of this study have to be seen in light of some limitations. First, the representativeness of the study sample is limited, as most relatives were female and of high education. Future studies should include a higher proportion of males and relatives from various educational backgrounds. Second, as findings are based on a cross-sectional survey, the generalizability may be limited, and further longitudinal studies are necessary.

Furthermore, a multidimensional measure based on Hebert et al. (2009) was used to assess preparedness for death. However, the overall scale showed only limited reliability, which could be because different dimensions of preparedness were measured. Future studies should develop a reliable and standardized scale to measure preparedness for death multidimensionally.

Because only two participants reported to be gender diverse, women and gender diverse participants were grouped together, therefore limiting generalizability of findings. Future studies should try to include a higher proportion of gender diverse people to explore possible differences in their psychological health compared to men and women.

Finally, this study focused on the experience of the forewarning period among relatives of people with cancer and did not include relatives of people with other illnesses. Future studies could include relatives of various illnesses and explore possible differences between them.

## Conclusions

The results show that pre-loss grief is a correlate for depressive, anxiety and somatization symptoms as well as satisfaction with life. Thus, the results suggest that people with high scores in pre-loss grief are particularly in need of early support. Furthermore, the results demonstrate that almost half of all subjects had moderate to severe depressive and anxiety symptoms. Interventions should therefore not only consider pre-loss grief, but also take the psychological health of relatives into account. In addition, because low levels of preparedness for death seem to be associated with greater somatization, interventions should address the various aspects of preparedness for death. Further studies are necessary to explore these relationships in different samples in a prospective manner.

## Supplemental Material

Supplemental Material - The Relationship Between Pre-Loss Grief, Preparedness and Psychological Health Outcomes in Relatives of People With Cancer Supplemental Material for The Relationship Between Pre-Loss Grief, Preparedness and Psychological Health Outcomes in Relatives of People With Cancer by Viktoria Schmidt, Julia Kaiser, Julia Treml, and Anette Kersting in OMEGA - Journal of Death and Dying
