# Supplementary material for: The Relationship Between Pre-Loss Grief, Preparedness and Psychological Health Outcomes in Relatives of People With Cancer
Source: Omega (Westport). 2022 Nov 24;91(2):798–810. doi: 10.1177/00302228221142675 (PMC12018713; doi:10.1177/00302228221142675)
Supplement: Supplemental Material - The Relationship Between Pre-Loss Grief, Preparedness and Psychological Health Outcomes in Relatives of People With Cancer [file sj-pdf-1-ome-10.1177_00302228221142675.pdf]

Supplementary Material.

S1. Self-generated questions for Preparedness for death.

	**If the sick person were to die soon,**				
		Not at all			Very much
1	... would you be emotionally prepared for this?	1	2	3	4
2	... would you already have all the information you need?	1	2	3	4
3	... would you be prepared for it organizationally (e.g., financially)?	1	2	3	4

Table S2. Comparison of Completers and Dropouts.

	Total sample (N=646)	Completers (N=299)	Dropouts (N=347)	p-value
Age, M (SD)	42.23 (17.84)	41.35 (12.21)	43.14 (22.22)	.231 [a]
Female or diverse, n (%)	517 (80.0)	272 (91.0)	245 (85.1)	.027 [b]
Married (%)	298 (46.1)	167 (55.9)	131 (37.8)	.180 [b]
School education				.083 [b]
Low	38 (5.9)	15 (5.0)	23 (6.5)	
Medium	180 (27.9)	93 (31.1)	87 (25.2)	
High	325 (50.3)	186 (62.2)	139 (40.1)	
Other	9 (1.4)	2 (0.7)	7 (2.0)	
Missing	94 (14.6)	3 (1.0)	91 (26.2)	
Religious, n (%)	385 (59.6)	174 (58.2)	211 (60.8)	.005 [b]
Relatives were				
Children	210 (32.5)	126 (42.1)	84 (24.2)	.011 [b]
Partners	114 (17.7)	80 (26.8)	34 (9.8)	
Parents	54 (8.4)	33 (11.0)	21 (6.1)	
Other	98 (3.5)	60 (20.1)	38 (11.0)	
Missing	170 (26.3)	-	170 (49.0)	

[a] two-tailored t-test, [b] χ^2 test

Table S3. Distribution of type of cancer of participants' loved ones.

Cancer type	n, %
Oral cavity and oropharyngeal cancer	14 (4.7)
Lung cancer	33 (11.1)
Gastrointestinal cancer	41 (13.7)
Bone cancer/ Chondrosarcoma	2 (0.7)
Melanoma	4 (1.3)
Cancer of soft tissues or mesothelioma	4 (1.3)
Breast cancer	54 (18.1)
Genital cancer	27 (9.0)
Cancer of the urinary system	7 (2.3)
Eye, brain & central nervous system cancer	41 (13.7)
Endocrine cancer	7 (2.3)
Hodgkin's lymphoma	5 (1.7)
Non-Hodgkin's lymphoma	7 (2.3)
Plasmocytoma	3 (1.1)
Leukemia	22 (7.4)
Multiple primary tumors	4 (1.3)
Not known	4 (1.3)
Other	20 (6.7)

Table S4. Subjective probability that the loved one will die within the next 5 years.

How likely do you think it is that the person with the disease will die within the next 5 years?	n (%)
0%	34 (11.3)
1-24%	43 (14.4)
25-49%	19 (6.4)
50%	42 (14.1)
51-74%	25 (8.4)
75-99%	55 (18.3)
100%	81 (27.1)
